# Conditional Survival With Increasing Duration of ICU Admission: An Observational Study of Three Intensive Care Databases

**DOI:** 10.1097/CCM.0000000000004082

**Published:** 2019-12-13

**Authors:** Dominic C. Marshall, Robert A. Hatch, Stephen Gerry, J. Duncan Young, Peter Watkinson

**Affiliations:** 1 Critical care research group, Nuffield Department of Clinical Neurosciences, University of Oxford, Oxford, United Kingdom.; 2 Oxford University Clinical Academic Graduate School, John Radcliffe Hospital, Oxford, United Kingdom.; 3 Centre for Statistics in Medicine, Nuffield Department of Orthopaedics, Rheumatology and Musculoskeletal Sciences, University of Oxford, Oxford, United Kingdom.

**Keywords:** conditional survival, length of stay, mortality, prolonged admission, survival

## Abstract

**Objectives:**

Prolonged admissions to an ICU are associated with high resource utilization and personal cost to the patient. Previous reports suggest increasing length of stay may be associated with poor outcomes. Conditional survival represents the probability of future survival after a defined period of treatment on an ICU providing a description of how prognosis evolves over time. Our objective was to describe conditional survival as length of ICU stay increased.

**Design:**

Retrospective observational cohort study of three large intensive care databases.

**Setting:**

Three intensive care databases, two in the United States (Medical Information Mart for Intensive Care III and electronic ICU) and one in United Kingdom (Post Intensive Care Risk-Adjusted Alerting and Monitoring).

**Patients:**

Index admissions to intensive care for patients 18 years or older.

**Interventions:**

None.

**Measurements and Main Results:**

A total of 11,648, 38,532, and 165,125 index admissions were analyzed from Post Intensive Care Risk-Adjusted Alerting and Monitoring, Medical Information Mart for Intensive Care III and electronic ICU databases respectively. In all three cohorts, conditional survival declined over the first 5–10 days after ICU admission and changed little thereafter. In patients greater than or equal to 75 years old conditional survival continued to decline with increasing length of stay.

**Conclusions:**

After an initial period of 5–10 days, probability of future survival does not decrease with increasing length of stay in unselected patients admitted to ICUs. These findings were consistent between the three populations and suggest that a prolonged admission to an ICU is not a reason for a pessimism in younger patients but may indicate a poor prognosis in the older population.

## Abstract 2

Supplemental Digital Content is available in the text.

Prolonged admissions to an ICU have an associated high resource utilization and personal cost to the patient. It is estimated that 23–45% of ICU bed days are occupied by long-stay patients (1). In a study of admissions to Canadian ICUs, those lasting more than 14 days constituted only 7.3% of admissions but accounted for 43.5% of ICU bed days (2). There is no consensus on what constitutes a prolonged intensive care admission and a range of definitions have been used. Previous descriptions of prolonged length of stay have ranged from 2 (3) to greater than 21 days (4). Previous studies have reported an association between total ICU length of stay and ICU and hospital mortality (5, 6). As a result, prolonged care on an ICU is sometimes viewed as an indicator of a slow response to treatment and hence poor prognosis. However, on any day of an ICU admission, there is uncertainty about the influence of the preceding length of stay on a patient’s ultimate outcome.

Conditional survival is defined as the probability of future survival, given that the patient has already survived for a certain period of time (7, 8). This has previously been used in studies of patients with cancer but not in those treated on an ICU. Applying these methods to patients treated in an ICU may provide useful information about how prognosis evolves over time, which can be factored into decision making about continuing therapy.

As progressively older and more comorbid patients are admitted to ICUs (9), it is important to understand how their response to a prolonged ICU admission differs from a younger cohort. For example, older and frail patients may lose muscle mass and power with increasing length of stay, while remaining exposed to the other risks of treatment on an ICU, reducing the probability of future survival. Conversely, they may take longer to respond to conventional therapy and longer to recover after their initial insult.

Despite accounting for a large proportion of bed days, the actual proportion of patients admitted to an ICU for an extended period is small (2). Large clinical databases therefore provide a cost-effective method of assessing conditional survival with sufficient power to analyze this cohort of patients. Using three unique intensive care databases, we compute conditional survival with increasing duration of ICU admission first in unselected patients and then in a subgroup analysis of the population dichotomized by age.

We hypothesized that the probability of future survival may change as duration of ICU admission increases and that this will not necessarily be a linear decrease in survival. Our primary objective is to assess probability of future survival with increasing duration of ICU admission.

## MATERIALS AND METHODS

### Data Source

We performed an observational study using three databases. The Post Intensive Care Risk-Adjusted Alerting and Monitoring (PICRAM) database contain details of all admissions between 2008 and 2016 to general ICUs in two English hospitals, a tertiary referral center and district general hospital. The Medical Information Mart for Intensive Care III (MIMIC-III) is an open-source clinical database, developed and maintained by Massachusetts Institute of Technology (MIT), Philips Healthcare, and Beth Israel Deaconess Medical Center (BIDMC) in the United States. Patients included in this database were admitted to one of the ICUs in BIDMIC between 2001 and 2012. The electronic ICU (eICU) collaborative database is composed of patients admitted to an ICU in the Philips telehealth program in the years 2014 and 2015. Two-hundred eight hospitals across continental United States contribute data to the database. The use of three independent databases allows us to limit bias that may exist within one dataset and further to demonstrate whether findings are consistent between the United Kingdom and North America, where different healthcare systems are in place and ICUs admit a different case-mix. Further, using MIMIC in addition to PICRAM extends the period of the study to 2001–2016.

### Patient Population

We included patients 18 years old or older at ICU admission with a record of vital status at hospital discharge. We only included data recorded during their first admission to ICU. We extracted age, sex, length of stay in an ICU, and survival to hospital discharge. Cohorts were dichotomized on age less than 75 or greater than or equal to 75 years old for an a priori planned subgroup analysis as 75 years has previously been used as cut off for “elderly” patients (10). The Oxford Acute Severity of Illness Score was used to describe severity of illness. This score is composed of 10 variables and has been demonstrated to have comparable predictive accuracy with other severity of illness score. It has previously been reported in Oxford data, MIMIC-III, and the eICU database (11).

The primary outcome of interest was survival to hospital discharge. Patients with missing data were excluded from the analysis. A flow chart of inclusion/exclusion criteria is included in **Supplementary Figure 1** (Supplemental Digital Content 1, http://links.lww.com/CCM/F27).

### Statistical Analysis

Simple descriptive statistics were calculated for each database and reported as number and percentage for binary variables and median and interquartile range (IQR) for continuous variables. To assess the main objective we calculated conditional survival, which we define as the proportion of patients surviving to hospital discharge given they have already survived a certain period of time on the ICU. We calculated this for each day of admission from zero until the day where fewer than 50 patients reached that length of stay (due to increasingly large CI widths after this day). The result at day zero is equivalent to the average hospital survival probability for all ICU admissions. We were able to use simple binomial methods rather than survival methods since discharge status was known for all patients, and therefore, no censoring occurred. We plotted survival to discharge against increasing length of stay and visually inspected for changes in the probability of future survival. We repeated this analysis with age subgroups (< 75 and ≥ 75 yr old).

We generated binomial CIs for conditional survival using the method by Agresti and Coull (12). Graphs were smoothed using local estimated scatterplot smooth (LOESS) with a span of 0.5. LOESS is a nonparametric smoothing procedure using a locally weighted least squares method to correct for influence of outliers and obtain a robust estimate of the trends. All statistical analysis was performed in R Core v3.4.4 (R: A Language and Environment for Statistical Computing, Vienna, Austria). Additional packages used were dplyr (version 0.8.1; https://cran.r-project.org/package=dplyr) and binom (version 1.1-1; https://cran.r-project.org/package=binom)

This study was conducted following STROBE guidelines and a checklist of recommendations and evidence is included in the **supplementary table** (Supplemental Digital Content 2, http://links.lww.com/CCM/F28) (16).

### Ethical Approval

PICRAM 1 was approved by the NRES Committee Oxford C on December 28, 2011 (ref: 11/SC/0440) and by the National Information Governance Board on February 2, 2012 (ref: ECC 7-05(f)/2011). The MIMIC-III and eICU databases have received ethical approval from the Institutional Review Boards (IRBs) at BIDMC and MIT and because the database does not contain protected health information, a waiver for the requirement for informed consent was included in the IRB approval.

## RESULTS

### PICRAM Database

**Figure 1.**
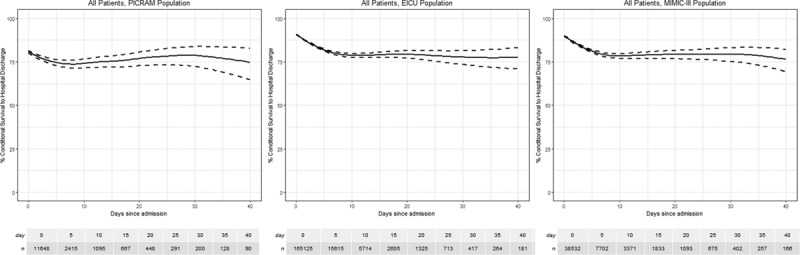
Conditional survival—survival to hospital discharge on each day of ICU admission for Post Intensive Care Risk-Adjusted Alerting and Monitoring (PICRAM) (*left*), Medical Information Mart for Intensive Care III (MIMIC-III) (*middle*), and electronic ICU (eICU) (*right*) populations. *Dashed lines* represent 95% CIs.

Eleven-thousand six-hundred forty-eight index admissions were included in the study after 524 were excluded for missing data (4.3%). Median age was 64 years (IQR, 50–74 yr), 58.7% were male, median length of stay was 1.9 days (IQR, 1.0–4.3 d). Hospital mortality was 18.5% (81.5% survival to hospital discharge) (**Table 1**). Ninety patients were admitted for longer than 40 days. Conditional survival declined over the initial 5–10 days but then plateaued prior to day 10 (**Fig. 1**). Conditional survival with a length of stay greater than 9 days declined to 74.2% (CI, 71.7–77.3%), at which point changes in conditional survival plateau with increasing length of stay.

**TABLE 1.**
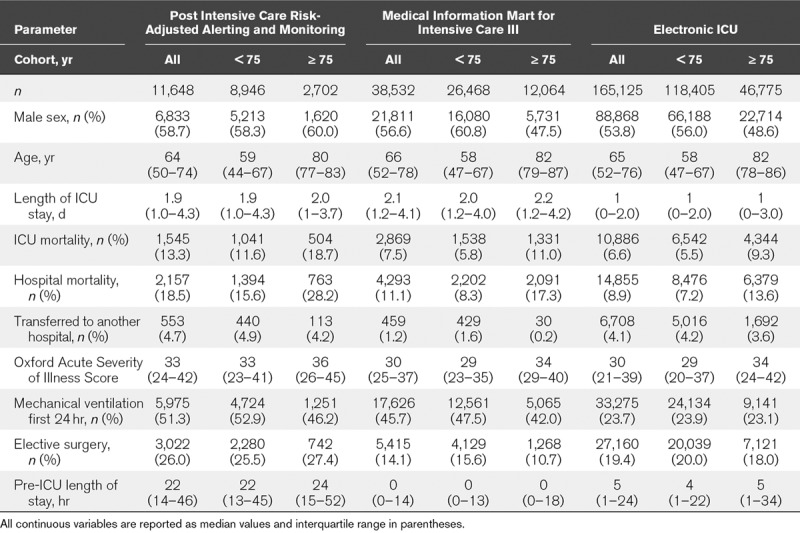
Descriptive Statistics for Three ICU Populations, Unselected and Then Dichotomized by Age

**Figure 2.**
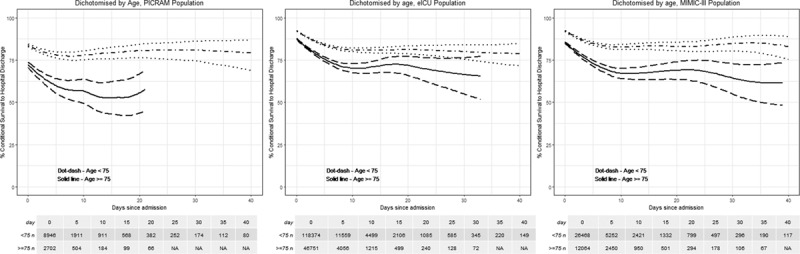
Conditional survival—survival to hospital discharge on each day of ICU admission stay for Post Intensive Care Risk-Adjusted Alerting and Monitoring (PICRAM) (*left*), Medical Information Mart for Intensive Care III (MIMIC-III) (*middle*), and electronic ICU (eICU) (*right*) populations dichotomized by age. *Dot-dash line* represents patients less than 75 yr old (*dotted lines* represent 95% CIs); *solid line* represents patients greater than or equal to 75 yr old (*dashed lines* represent 95% CIs).

When patients were dichotomized into less than 75 years old and greater than or equal to 75, there was an overall reduced survival from initial admission in the older age group, which was followed by a steeper decrease in conditional survival with increasing length of stay (**Fig. 2**). Fewer than 50 patients over the age of 75 years had durations of stay longer than 21 days in ICU. The probability of future survival continued to decrease up to ~15 days in the older cohort with 52.3% (CI, 43.0–61.4%) conditional survival with duration of stay greater than 4 days. This was contrasted with 79.2% (CI, 75.8–82.2%) at the same time point in the younger cohort.

### MIMIC-III

Thirty-eight–thousand five-hundred thirty-two index admissions were included in the initial cohort, no admissions were excluded for missing data, median age was 66 years (IQR, 52–78 yr), 56.6% were male, median length of stay was 2 days (IQR, 1–4 d), and 88.9% survived to hospital discharge (Table 1). This initial cohort reduced to 166 patients who were admitted for longer than 40 days. In all patients, conditional survival declined over the initial 10 days but then plateaued with minimal changes in conditional survival as length of stay increased (Fig. 1). Initial survival of 88.9% (CI, 88.5–89.2%) for all patients admitted to the ICU declined to a conditional survival of 78.8% (CI, 77.4–80.0%) in patients admitted for greater than 9 days and remained at this level (supplementary table, Supplemental Digital Content 1, http://links.lww.com/CCM/F27).

When patients were dichotomized by age, there was a steeper decrease in conditional survival in the older age group with increased length of stay (Fig. 2). The probability of hospital survival was lower at admission in older patients, 82.7% (CI, 82–83.3%) compared with 91.7% (CI, 91.3–92.0%). Conditional survival continued to decrease in the older population to 68.0% (CI, 65.2–70.1%) in patients admitted for greater than 9 days before plateauing (supplementary table, Supplemental Digital Content 1, http://links.lww.com/CCM/F27).

### eICU

One-hundred sixty-five–thousand one-hundred twenty-five index admissions were included in the initial cohort, 1,696 admissions were excluded due to missing data (1%), median age was 65 years (IQR, 52–76 yr), 53.8% were male, median length of stay was 1 day (IQR, 0–2 d) and 91.0% survived to hospital discharge (Table 1). This initial cohort reduced to 181 patients who were admitted for longer than 40 days. Conditional survival declined over the initial 10 days but then plateaued with minimal changes in as length of stay increased (Fig. 1). Conditional survival at admission decreased from 90.3% to 84.2% in patients admitted to ICU for greater than 9 days.

When patients were dichotomized by age, survival at admission was 85.9% in the older cohort compared with 92.8% in the younger cohort and was followed by a steeper decrease in conditional survival in the older age group (supplementary table, Supplemental Digital Content 1, http://links.lww.com/CCM/F27). Although the initial decline was greater in the older population, the conditional survival plateaued at a similar time point of around 10 days of ICU (Fig. 2).

#### Comparison of Databases

The median ages of the patients in each database were similar, median length of stay was shorter in the eICU database (Table 1). Hospital mortality rates were higher in the PICRAM database, 18.5% compared with 11.1% and 9.0% in MIMIC-III and eICU, respectively. Median OASIS scores were also higher in PICRAM, 33 compared with 30 in MIMIC and eICU. Patients in the eICU databases had lower rates of mechanical ventilation compared with PICRAM and OASIS.

## DISCUSSION

In an observational retrospective study of three intensive care databases, we have demonstrated that in unselected patients admitted to an ICU, after an initial period of 5–10 days conditional survival to hospital discharge does not decrease with length of ICU stay. Assessment of conditional survival generates prognostic information in how the probability of surviving to hospital discharge changes as length of ICU stay increases. When the populations were dichotomized by age, less than 75 years, and greater than or equal to 75 years, this finding persisted in the younger cohorts, but in the older cohorts, conditional survival decreased with increasing length of stay for a longer period and also declined more sharply.

To our knowledge, this is the first report to assess ICU mortality as conditional survival with the aim of describing changing mortality over the duration of an ICU admission. This method has previously been used in oncology to describe changing hazard over time and describes the probability of ongoing survival if a patient has survived to a point in time (7). Previous studies have used regression methods to assess how a patient’s total length of stay is linked to their probability of survival. However, here we describe the probability of future survival given they have already survived a certain period of time.

The mortality rate is higher in the PICRAM database of English ICUs which is possibly due to these ICUs admitting patients with higher illness severity scores as has previously been reported (14). However, the OASIS score does not differ as significantly as might be expected considering the difference in mortality which is approximately double in PICRAM compared with the eICU database. The OASIS score only uses 10 variables, and it is likely that it does not account fully for the differences in severity of illness between databases. Further, if eICU contains a significantly different case-mix from the conventional ICUs used to develop and validate the score, it may perform less well. The eICU population has a shorter length of stay than MIMIC-III and PICRAM, median length of stay 1 day contrasted with 2 days in the other populations. This may represent more complex patients being transferred to tertiary referral centers. However, it should be noted that patients in all three databases have a relatively short median length of stay. PICRAM also contains a high proportion of elective surgical patients which is associated with lower mortality. With bed pressures in the United Kingdom, it is likely that only the most high-risk surgical procedures involve an elective ICU admission. Finally, it is noted that PICRAM has the highest proportion of intubated patients at admission. Intubation is associated with high mortality and is included with the OASIS score.

There is no consensus on what constitutes a “prolonged stay” in an ICU. However, attempts have been made to characterize a subpopulation of patients with “persistent critical illness” (15). This occurs when the admitting diagnosis is no longer the reason for their continued treatment on an ICU (16). Previous studies in Scotland and Australia have characterized patients with very long ICU admissions of greater than 30 or greater than 60 days and have shown that many survive (17, 18). However, with a median duration of ICU stay of 2 days (IQR, 1–5 d) (19), it may be that a much shorter duration of ICU admission could be considered prolonged. In all three cohorts assessed, there appears to be an initial phase of worsening probability of future survival with increasing length of stay. However, this plateaued within 10 days with little change in conditional survival after this time. Our results suggest that the change in phenotype to that of long stayer may occur as early as 10 days. In the PICRAM population, there was a favorable trend after 15 days not seen in the other populations, it is unclear whether this is a true effect or variance from low patient numbers.

Our findings of an initial period of reducing survival with increased stay is supported by the findings of Williams et al (20) who report in an Australian ICU population a linear decrease in long term mortality for an initial 5-day period followed by a relatively constant risk. Other reports have also demonstrated favorable outcomes in patients with prolonged admissions to ICU (17, 21). This information is useful for clinicians and patients because it highlights that a prolonged or increasing length of stay is not a reason for pessimism. It may be that a belief that increasing duration of stay confers a poor prognosis contributing to the sharper decline in the older population where life-sustaining measures are being withdrawn.

In patients greater than or equal to 75 years old their survival at admission is lower than a younger cohort and their conditional survival declines more rapidly and plateaus after a longer duration of ICU admission. In the PICRAM population, only conditional survival appeared to improve in the older population around day 15, however, the number of patients at this time point is small and the significance of this trend is unclear. The finding that older patients have worse outcomes has been highlighted in previous reports (10) and age is usually a component of illness severity scoring systems such as Acute Physiology and Chronic Health Evaluation II. However, the difference in response to increasing duration of stay is novel. The impact of age on probability of future survival may, in part, be due to a reduction in physiologic reserve and faster rate of deconditioning that occurs with aging. Older patients have been demonstrated to decondition within the first 24 hours of hospital admission (22). A prospective analysis of patients greater than 80 years old admitted to Canadian ICUs demonstrated a poor functional recovery and high mortality rate (23). However, our retrospective analysis, cannot correct for any selection bias that may be resulting from the medical team withdrawing care earlier in elderly patients.

This study has a number of important limitations. As duration of ICU admission increases, there are fewer patients remaining within the cohort to assess conditional survival. To minimize any bias, this may introduce we a priori set an arbitrary cutoff of a minimum of 50 patients for continued analysis. However, as length of stay increases, the precision in our findings diminishes as represented by widening error bars on Figs. 1 and 2. The limited cohort size for these more extended stays indicates the findings may not be generalizable to all ICU patients with prolonged admissions. As this is an observational study, we cannot account for any impact of length of stay on decisions to continue or withdraw care which may have an impact on future survival. Further, a proportion of patients will have had their care stepped down from level 3 to level 2 care or alternatively may have delayed discharge to the ward. We have been unable to account for this in our analysis. We have included within our analysis a minority of patients who have been transferred out either to an ICU or a ward at an alternative hospital. A proportion of these patients may die prior to discharge for the same episode of illness, these events would not be captured and contribute to our primary outcome.

The strengths of this study include a large population of unselected ICU patients, which provides good generalizability. Further, we have replicated the findings in two North American ICU populations and demonstrated consistent findings. That these findings remain consistent despite the differences between the ICU populations lends considerable support to the validity of our findings. Large clinical datasets are limited by their reliability of data collection and assumptions that are required to construct the database. Therefore, we have limited our study to reporting objective measures, specifically age, sex, length of stay, ICU, and hospital mortality. In PICRAM and eICU, outcome data were not available for 4% and 1% of the cohorts, respectively. Exclusion of these subjects may have biased our findings toward higher or lower hospital survival, but the effect would be limited given the small exclusion rates. Further work may focus on case-mix adjustment to assess the impact of initial and evolving severity of illness scores on conditional survival. Additionally, analysis of subpopulations of patients with different diagnoses may yield contrasting patterns of conditional survival and further inform clinicians when assessing the impact of length of stay on patient outcome.

## CONCLUSIONS

After an initial period of 5–10 days, probability of future survival does appear to decrease with increasing length of stay in unselected patients admitted to ICUs in United Kingdom and United States. Length of stay in itself should therefore not be factored in to decisions around withdrawal of life-sustaining measures. In a subpopulation of patients, 75 years or older probability of future survival continued to decrease with increasing length of stay.

## Supplementary Material
