# Supplementary material for: Conditional Survival With Increasing Duration of ICU Admission: An Observational Study of Three Intensive Care Databases
Source: Crit Care Med. 2019 Dec 13;48(1):91–7. doi: 10.1097/CCM.0000000000004082 (PMC6919217; doi:10.1097/CCM.0000000000004082)
Supplement: Supplementary file 1 [file ccm-48-091-s001.docx]

**Supplementary materials**

**
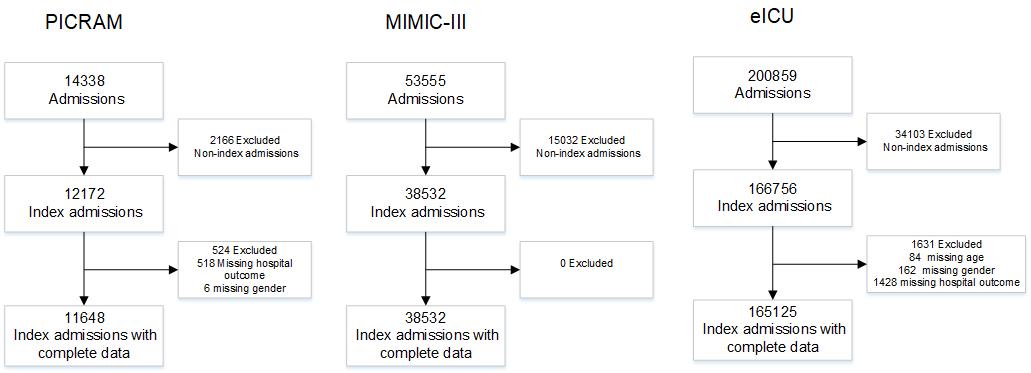
**

*Supplementary Figure 1: Flow chart of inclusion/exclusion criteria for PICRAM, MIMIC-III and eICU*

*Supplementary Table 1****:*** *Conditional survival (survival to hospital discharge from this point in time) as percentage for three ICU populations, unselected and then dichotomised by age. *Indicates fewer than 50 patients in cohort*

|  | PICRAM | | | MIMIC-III | | | eICU | | |
| --- | --- | --- | --- | --- | --- | --- | --- | --- | --- |
| Cohort (years) | All | < 75 | ≥ 75 | All | < 75 | ≥ 75 | All | < 75 | ≥ 75 |
| All patients | 81.5 | 84.4 | 71.8 | 88.9 | 91.7 | 82.7 | 91.0 | 92.8 | 86.4 |
| >4 days | 73.8 | 77.1 | 61.4 | 81.6 | 84.8 | 75 | 84.2 | 85.9 | 79.7 |
| >9 days | 74.2 | 78.0 | 56.3 | 78.8 | 83.1 | 68 | 78.8 | 81.2 | 70.6 |
| >14 days | 75.2 | 79.2 | 52.3 | 78.4 | 82.8 | 66.9 | 78.6 | 80.7 | 70.2 |
| >19 days | 76.2 | 80.2 | 52.9 | 79.8 | 83.7 | 69.4 | 79.4 | 80.6 | 73.4 |
| >24 days | 78.2 | 81.0 | * | 79.9 | 83.7 | 69.5 | 78.8 | 80.9 | 69.5 |
| >29 days | 79.2 | 81.1 | * | 79.9 | 85.6 | 64.1 | 77.8 | 80.1 | 66.7 |
| >34 days | 77.5 | 80.2 | * | 79.3 | 85.2 | 63.7 | 77.1 | 79.2 | * |
| >39 days | 75.8 | 80.0 | * | 76.2 | 82.2 | 62.5 | 76.6 | 77.6 | * |
