# Supplementary material for: Conditional Survival With Increasing Duration of ICU Admission: An Observational Study of Three Intensive Care Databases
Source: Crit Care Med. 2019 Dec 13;48(1):91–7. doi: 10.1097/CCM.0000000000004082 (PMC6919217; doi:10.1097/CCM.0000000000004082)
Supplement: Supplementary file 2 [file ccm-48-091-s002.docx]

| **Title and abstract** | | | **Present? Details** | **Location** |
| --- | --- | --- | --- | --- |
|  | 1 | (*a*) Indicate the study's design with a commonly used term in the title or the abstract | Yes | Page 1 |
|  |  | (*b*) Provide in the abstract an informative and balanced summary of what was done and what was found | Yes | Page 2 |
| **Introduction** | | |  |  |
| Background/rationale | 2 | Explain the scientific background and rationale for the investigation being reported | Yes | Page 3 |
| Objectives | 3 | State specific objectives, including any prespecified hypotheses | Yes | Page 4, final paragraph of introduction |
| **Methods** | | |  |  |
| Study design | 4 | Present key elements of study design early in the paper | Yes | Study design in penultimate paragraph of introduction and first paragraph of methods. |
| Setting | 5 | Describe the setting, locations, and relevant dates, including periods of recruitment, exposure, follow-up, and data collection | Yes | First paragraph of methods, *data source* section. |
| Participants | 6 | (*a*) *Cohort study*?Give the eligibility criteria, and the sources and methods of selection of participants. Describe methods of follow-up*Case-control study*?Give the eligibility criteria, and the sources and methods of case ascertainment and control selection. Give the rationale for the choice of cases and controls*Cross sectional study*?Give the eligibility criteria, and the sources and methods of selection of participants | Inclusion and exclusion criteria clearly defined. | Second paragraph methods, *patient population* section. |
|  |  | (*b*) *Cohort study*?For matched studies, give matching criteria and number of exposed and unexposed*Case-control study*?For matched studies, give matching criteria and the number of controls per case | No matching | NA |
| Variables | 7 | Clearly define all outcomes, exposures, predictors, potential confounders, and effect modifiers. Give diagnostic criteria, if applicable | Yes | *Patient population* and *outcomes* section of method. |
| Data sources/ measurement | 8* | For each variable of interest, give sources of data and details of methods of assessment (measurement). Describe comparability of assessment methods if there is more than one group | Yes | *Patient population* and *outcomes* section of method. |
| Bias | 9 | Describe any efforts to address potential sources of bias | Yes | Described in methods and limitations section of discussion. |
| Study size | 10 | Explain how the study size was arrived at | Yes | Reported in brief in the results and flow diagram of how cohort was derived included as supplementary materials. |
| Quantitative variables | 11 | Explain how quantitative variables were handled in the analyses. If applicable, describe which groupings were chosen and why | Yes | Described in *statistical analysis* section of methods. |
| Statistical methods | 12 | (*a*) Describe all statistical methods, including those used to control for confounding | Yes - no control for confounding. | Described in *statistical analysis* section of methods. |
|  |  | (*b*) Describe any methods used to examine subgroups and interactions | Yes | *Patient population* section of methods. |
|  |  | (*c*) Explain how missing data were addressed | Yes – records removed. | Demonstrated in flow diagram representing how cohort was derived in supplementary materials. |
|  |  | (*d*) *Cohort study*?If applicable, explain how loss to follow-up was addressed*Case-control study*?If applicable, explain how matching of cases and controls was addressed*Cross sectional study*?If applicable, describe analytical methods taking account of sampling strategy | No loss to follow up occurred as outcome was at hospital discharge. However there are missing data for hospital survival as described above. |  |
|  |  | (*e*) Describe any sensitivity analyses | Yes | The MIMIC-III and eICU population represent a sensitivity analysis and as described in methods the analysis is identical as performed for PICRAM database. |
| **Results** | | |  |  |
| Participants | 13* | (*a*) Report numbers of individuals at each stage of study?eg numbers potentially eligible, examined for eligibility, confirmed eligible, included in the study, completing follow-up, and analysed | Yes | Reported in results and in detail in the supplementary materials |
|  |  | (*b*) Give reasons for non-participation at each stage | Yes | Flow diagram |
|  |  | (*c*) Consider use of a flow diagram | Yes | Supplementary materials. |
| Descriptive data | 14* | (*a*)Give characteristics of study participants (eg demographic, clinical, social) and information on exposures and potential confounders | Yes | Table 1 |
|  |  | (*b*) Indicate number of participants with missing data for each variable of interest | Yes | Flow diagram |
|  |  | (*c*) *Cohort study*?Summarise follow-up time (eg average and total amount) | Yes | Follow up time was survival to discharge which is reported. |
| Outcome data | 15* | *Cohort study*?Report numbers of outcome events or summary measures over time | *Yes* | Table 1 |
|  |  | *Case-control study?*Report numbers in each exposure category, or summary measures of exposure | Na |  |
|  |  | *Cross sectional study?*Report numbers of outcome events or summary measures | *NA* |  |
| Main results | 16 | (*a*) Report the numbers of individuals at each stage of the study?eg numbers potentially eligible, examined for eligibility, confirmed eligible, included in the study, completing follow-up, and analysed | Yes – supplementary materials | Flow diagram |
|  |  | (*b*) Give reasons for non-participation at each stage | Yes | Flow digram |
|  |  | (*c*) Consider use of a flow diagram | Yes | Flow diagram in supplementary materials |
| Other analyses | 17 | Report other analyses done?eg analyses of subgroups and interactions, and sensitivity analyses | Yes – subgroup analysis of < 75 years of age and > 75 years of age. |  |
| **Discussion** | | |  |  |
| Key results | 18 | Summarise key results with reference to study objectives | Yes | First paragraph of discussion. |
| Limitations | 19 | Discuss limitations of the study, taking into account sources of potential bias or imprecision. Discuss both direction and magnitude of any potential bias | Yes | Penultimate paragraph, *limitations* section. |
| Interpretation | 20 | Give a cautious overall interpretation of results considering objectives, limitations, multiplicity of analyses, results from similar studies, and other relevant evidence | Yes |  |
| Generalisability | 21 | Discuss the generalisability (external validity) of the study results | Yes | Discussed in strengths of study |
| **Other information** | | |  |  |
| Funding | 22 | Give the source of funding and the role of the funders for the present study and, if applicable, for the original study on which the present article is based | Yes | Page 2 |
